# Health Care Interventions Delivered Over the Internet: How Systematic was the Review? - Author's Reply

**DOI:** 10.2196/jmir.8.2.e12

**Published:** 2006-06-30

**Authors:** Frances Griffiths

## Author's Response

We thank Evan Mayo-Wilson for raising the issue on how systematic and exhaustive our search for our recent qualitative analysis [1] was. This was not a systematic review as in a common usage of the term for example by the Cochrane Collaboration. We used systematic methods to undertake a qualitative review of the literature on health care interventions delivered over the Internet. To identify common themes it was important to identify a broad range of published studies but we did not feel that it was necessary to be exhaustive. In our paper we describe in some detail how we identified the literature including the use of three existing systematic reviews, a hand search of JMIR and our own previously published literature review. Through the triangulation of these search approaches we aimed to identify the main body of relevant literature. We realise we may not have identified every published paper of relevance.

Thank you for drawing our attention to the paper by Klein and Richards [2]. This paper would be excluded from our review. As mentioned in our paper the focus of our review was interventions where the networking provided by the Internet is a component of the intervention. One of our exclusion criteria was “no networked features, such as computer-based decision support systems delivered from a CD or interventions where there was no use of the Internet beyond delivery (ie, they could have been delivered by a CD)”. From the description of the intervention in the Klein and Richards paper it appears to have no networked features.
